# Evaluating Therapeutic Plasma Exchange in Pediatric Acute Disseminated Encephalomyelitis: A Comprehensive Review

**DOI:** 10.7759/cureus.64190

**Published:** 2024-07-09

**Authors:** Tanvi Bhardwaj, Sunil Kumar, Neha Parashar, Gyaneshwar Tiwari, K.M. Hiwale

**Affiliations:** 1 Pathology, Jawaharlal Nehru Medical College, Datta Meghe Institute of Higher Education and Research, Wardha, IND; 2 Pathology, Lok Nayak Hospital, Delhi, IND

**Keywords:** treatment, neuroinflammation, autoimmune, therapeutic plasma exchange (tpe), pediatric, acute disseminated encephalomyelitis (adem)

## Abstract

Acute disseminated encephalomyelitis (ADEM) is a rare autoimmune disorder characterized by brain and spinal cord inflammation. In pediatric patients, ADEM presents unique challenges due to its potential for rapid progression and long-term neurological sequelae. Therapeutic plasma exchange (TPE) has emerged as a potential treatment option by targeting the underlying autoimmune process and modulating the inflammatory response. This comprehensive review evaluates the role of TPE in pediatric ADEM, synthesizing evidence from clinical studies and providing insights into its efficacy, safety, and potential benefits. The review highlights the variability in TPE efficacy based on disease severity and patient-specific factors. Implications for clinical practice include considering TPE as a therapeutic option, particularly in severe or refractory cases, and emphasizing the importance of early intervention. Recommendations for future research include long-term prospective studies, comparative effectiveness trials, and efforts to standardize TPE protocols. Overall, continued investigation and innovation in managing pediatric ADEM are essential for improving outcomes and quality of life for affected children and their families.

## Introduction and background

Acute disseminated encephalomyelitis (ADEM) is a rare autoimmune disease characterized by brain and spinal cord inflammation. It typically follows viral infections or vaccinations and presents with a wide range of neurological symptoms, including encephalopathy, motor deficits, and demyelination [1]. In pediatric patients, ADEM poses significant challenges due to its potential for rapid progression and long-term neurological sequelae. Evaluating treatment options in this population is crucial for optimizing outcomes, minimizing disability, and improving quality of life [2].

Therapeutic plasma exchange (TPE), also known as plasma exchange or plasmapheresis, is a medical procedure that involves removing plasma from the blood and replacing it with a replacement solution. TPE has emerged as a potential therapeutic intervention for ADEM by targeting the underlying autoimmune process and modulating the inflammatory response [3]. This comprehensive review aims to critically evaluate the role of TPE in managing pediatric ADEM. By synthesizing evidence from clinical studies and analyzing outcomes, the review seeks to provide insights into TPE's efficacy, safety, and potential benefits as a treatment option for pediatric patients with ADEM.

## Review

Pathophysiology of ADEM

Immunological Mechanisms Underlying ADEM

ADEM is believed to involve several immunological mechanisms contributing to its pathogenesis. One such mechanism is molecular mimicry, where the immune system erroneously targets myelin due to similarities between viral or bacterial antigens and self-antigens within the central nervous system (CNS) [4,5]. This process triggers an autoimmune response against the myelin sheath, leading to demyelination and subsequent neurological symptoms. Another mechanism in ADEM is epitope spreading, wherein the immune response diversifies to include additional self-antigens beyond the initial target. This broader autoimmune reaction against CNS components exacerbates tissue damage and contributes to the progression of the disease [5]. Bystander activation is also observed in ADEM, wherein an infection nonspecifically triggers the immune response. Although not directly targeting myelin, this activation of immune cells can still contribute to tissue damage in the CNS, further exacerbating the inflammatory response and neurological symptoms [4]. The hallmark of ADEM is its autoimmune inflammatory response within the CNS, characterized by the attack of immune cells on the myelin sheath. This attack leads to inflammatory demyelinating brain and spinal cord lesions, disrupting nerve signal transmission and manifesting clinically as neurological symptoms [6,7]. The interplay of these immunological mechanisms underscores the complex pathophysiology of ADEM. It highlights the importance of targeted therapeutic interventions to modulate the autoimmune response and mitigate tissue damage in affected individuals.

Neurological Manifestations and Complications

**Figure 1:**
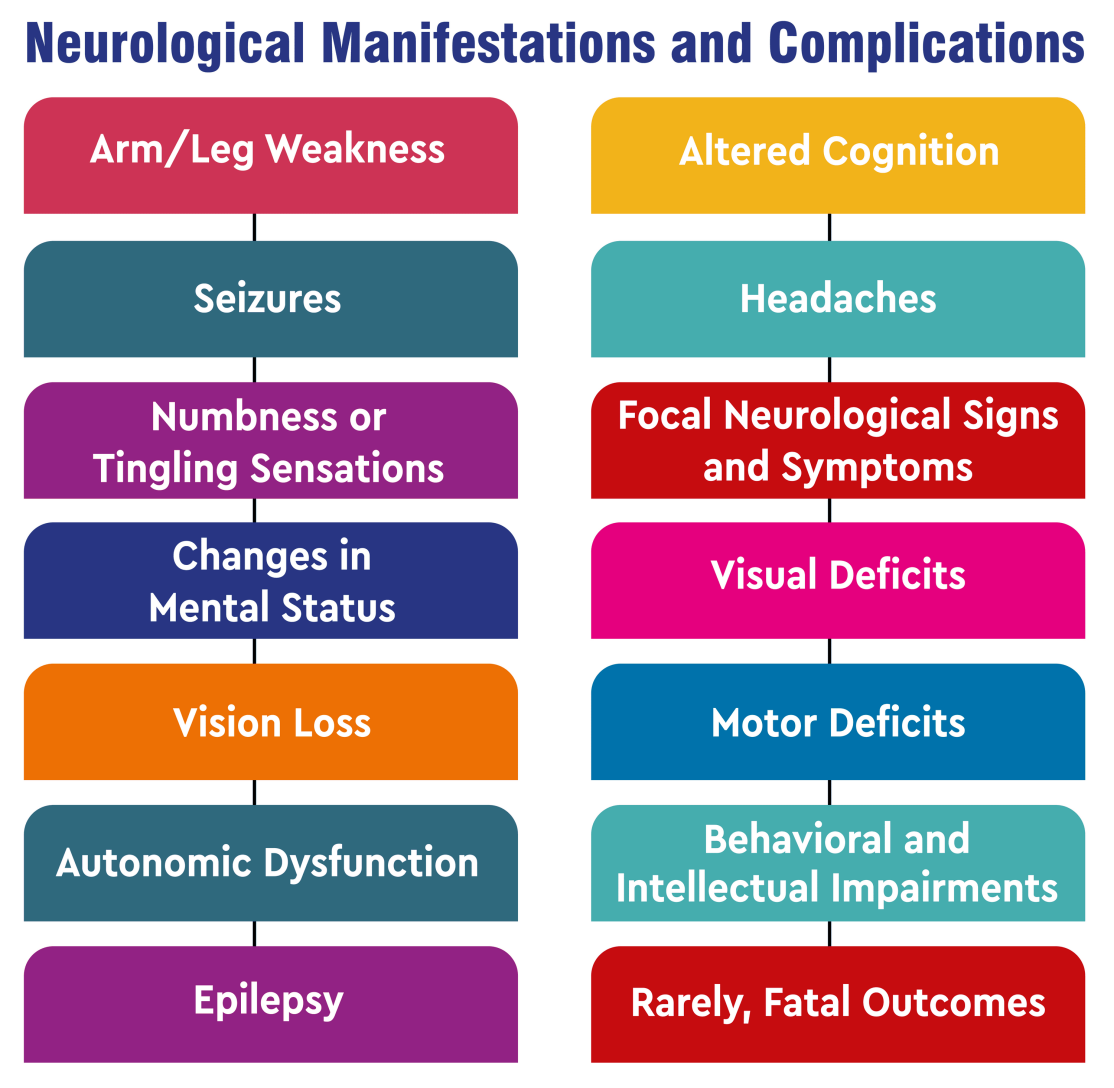
Neurological manifestations and complications The image is created by the corresponding author.

The neurological manifestations of ADEM encompass a spectrum of symptoms, including arm and leg weakness, seizures, numbness or tingling sensations, alterations in mental status, vision impairment, changes in cognition, headaches, and focal neurological signs and symptoms [7-9]. These symptoms exhibit variability in severity and may be accompanied by complications affecting various aspects of neurological function. Complications associated with ADEM encompass deficits in visual perception, motor function, autonomic regulation, and behavioral or intellectual capacities, along with the development of epilepsy. In exceedingly rare instances, ADEM can culminate in fatal outcomes [2]. Moreover, some patients may experience persistent symptoms following treatment, resulting in residual focal neurological deficits in a subset of cases [2]. The array of neurological manifestations and complications associated with ADEM underscores the multifaceted impact this disorder can exert on the CNS, spanning from physical impairments to cognitive and behavioral challenges. Timely recognition, accurate diagnosis, and appropriate management are imperative in addressing these manifestations and mitigating potential long-term complications [1]. Figure 1 shows neurological manifestations and complications.

Diagnostic Criteria and Challenges

ADEM presents distinctive clinical features characterized by monophasic encephalopathy alongside polyfocal neurological symptoms. The initial prodromal phase exhibits considerable variability and nonspecificity, often manifesting with symptoms such as fever, headache, and nausea. Encephalopathy, a pivotal diagnostic indicator, may manifest subtly as alterations in behavior, somnolence, or irritability, particularly prevalent among pediatric patients [10,11]. Imaging findings, particularly magnetic resonance imaging (MRI) of the brain, play a pivotal role in ADEM diagnosis. Acute signs of extensive, multifocal, and subcortical white matter abnormalities on MRI indicate demyelination within the CNS, hallmark features of ADEM [7,11]. ADEM's cerebrospinal fluid (CSF) analysis often reveals nonspecific lymphocytic pleocytosis and elevated albumin levels. Although not consistently present, transient detection of oligoclonal bands may occur. These CSF findings and clinical and imaging data contribute significantly to diagnosing ADEM [7,11]. ADEM must be meticulously differentiated from other conditions, particularly multiple sclerosis (MS). The distinction between monophasic ADEM and the initial attack of MS holds crucial clinical significance. While no specific biological marker exists for ADEM, a comprehensive assessment combining clinical, imaging, and laboratory findings aids in distinguishing between these conditions [11]. Challenges in ADEM diagnosis stem from the variability of initial symptoms, necessitating heightened clinical suspicion and the potential for misdiagnosis. Timely diagnosis is paramount for facilitating early intervention and achieving favorable prognostic outcomes in ADEM, as delays in diagnosis may result in unnecessary medical interventions and increased healthcare costs [10,11].

TPE

Historical Context and Development

TPE boasts a rich history as a treatment modality, dating back well over a century. Its inaugural application dates to 1914, when Abel, Rowntree, and Turner pioneered plasmapheresis procedures on canines [12]. By 1952, TPE had already found utility in managing hyperviscosity in patients with multiple myeloma [12]. The 1960s witnessed significant technological strides in TPE, exemplified by Schwab and Fahey's successful 1960 procedure to lower high globulin levels in a macroglobulinemia patient [13]. This pivotal moment marked the commencement of TPE's widespread adoption across diverse medical domains. By the 1970s, TPE had emerged as a viable treatment avenue for myriad neurological disorders [12]. The American Society for Apheresis (ASFA) further cemented TPE's standing in medical practice by furnishing guidelines for its application across various conditions [12]. TPE is a firmly entrenched treatment approach for an extensive spectrum of ailments, spanning blood disorders, neurological maladies, and autoimmune conditions [14]. It is lauded for its safety, cost-effectiveness, and life-saving potential [12]. The ASFA currently advocates for TPE in managing 87 distinct diseases, encompassing 179 indications [12].

Mechanism of Action

TPE operates through several mechanisms to exert its therapeutic effects in various autoimmune and neuroimmunological disorders. Firstly, TPE facilitates the removal of pathological substances, such as autoantibodies, immune complexes, and monoclonal paraproteins, from the plasma, all of which contribute to disease pathogenesis [15]. By eliminating these aberrant components, TPE helps mitigate disease activity and progression. Secondly, TPE replenishes deficient plasma components by utilizing plasma as a replacement fluid. This process aids in restoring normal levels of beneficial plasma proteins and factors, thus promoting physiological homeostasis [15]. Furthermore, TPE can induce alterations in lymphocyte proliferation and function. TPE may enhance the efficacy of these therapeutic interventions by sensitizing lymphocytes to the effects of immunosuppressants and chemotherapeutic agents [15]. Additionally, TPE exhibits immunomodulatory effects by modulating B and T cell numbers and activation, augmenting T suppressor function, and altering the T-helper cell type 1/2 (Th1/Th2) ratio [15]. These immunomodulatory actions contribute to the regulation of immune responses and the attenuation of autoimmune activity. Moreover, TPE reduces inflammatory mediators, such as cytokines and chemokines, from the plasma, which dampens inflammation [15]. This anti-inflammatory effect is instrumental in alleviating symptoms and mitigating tissue damage associated with autoimmune and neuroimmunological disorders. While the precise mechanisms underlying the therapeutic efficacy of TPE may vary depending on the specific disease, these multifaceted effects likely play a pivotal role in its therapeutic benefits. However, further research is warranted to fully elucidate the intricate mechanisms driving the therapeutic actions of TPE.

Indications and Contraindications

TPE treats diverse autoimmune, neurological, and hematological conditions. Within the neurology domain, TPE has demonstrated efficacy in managing ailments like myasthenia gravis (MG) (for short-term acute care), chronic inflammatory demyelinating polyneuropathy, and acute CNS demyelination disorders unresponsive to steroids, encompassing MS and ADEM. Moreover, TPE finds application in addressing hyperviscosity linked with hypergammaglobulinemia, thrombotic thrombocytopenic purpura (TTP), and Guillain-Barré syndrome (GBS) [16]. Recently, investigations have explored TPE's potential as a therapeutic avenue for neuromyelitis optica spectrum disorder (NMOSD). Nonetheless, TPE is not devoid of contraindications. Patients lacking central line access or large bore peripheral lines, experiencing hemodynamic instability, septicemia, known allergies to fresh frozen plasma, replacement colloids, albumin, or heparin, exhibiting hypocalcemia, or having recently used angiotensin-converting enzyme (ACE) inhibitors are unsuitable candidates for TPE. Thoughtful patient selection and vigilant monitoring are imperative for TPE's safe and efficacious implementation in clinical settings. As ongoing research continues to delineate TPE's potential advantages in diverse neurological maladies, assessing indications vis-à-vis contraindications and potential risks remains crucial to ensure optimal patient outcomes [17].

Efficacy and Safety Profile

The efficacy and safety of TPE have been extensively scrutinized across diverse patient demographics, encompassing pediatric and adult populations. Research findings underscore TPE's effectiveness as a therapeutic avenue for select pediatric disorders, with studies underscoring its safety and efficacy in pediatric patient cohorts [18]. TPE's utility lies in its capacity to eliminate highly protein-bound toxins and substances characterized by delayed metabolic effects from the bloodstream, rendering it invaluable across a spectrum of medical conditions [19]. Regarding safety considerations, when administered by proficient healthcare personnel, TPE is deemed a safe and efficacious therapeutic intervention for critically ill patients afflicted with conditions outlined in the ASFA guidelines [3]. However, like any medical procedure, TPE entails inherent risks, including hypotension, dyspnea, metabolic alkalosis, hemorrhage, infection susceptibility, electrolyte imbalances, and allergic reactions in instances involving donor plasma utilization [3]. These potential risks underscore the imperative of diligent oversight by seasoned healthcare teams during TPE procedures, ensuring patient safety and fostering favorable outcomes. The extant body of evidence suggests that TPE is a pivotal treatment modality across various disease spectra, offering both efficacy in eliminating detrimental substances from the bloodstream and a commendable safety profile under judicious clinical supervision [3,18].

Review of clinical studies

Summary of Key Studies Evaluating TPE in Pediatric ADEM

A retrospective study examined pediatric cases of ADEM where TPE was employed as adjuvant therapy after inadequate response to steroids and intravenous immunoglobulin (IVIG). The findings indicated a progressive clinical amelioration among patients, characterized by enhanced responsiveness to stimuli, cessation of seizures, and restoration of mobility. Remarkably, most patients exhibited no new demyelinating episodes post-TPE intervention [20]. Additionally, a case report spotlighted a pediatric ADEM patient refractory to standard treatments, namely, steroids and IVIG, which demonstrated substantial improvement following TPE. This case underscored the tolerability of TPE and its association with neurological enhancement in pediatric ADEM scenarios [21]. Moreover, a study involving 10 pediatric patients afflicted with acute demyelinating syndromes, including ADEM, corroborated TPE's feasibility, efficacy, and safety. Notably, patients with ADEM showcased improvements in Expanded Disability Status Scale (EDSS) and Gait Scale (GS) scores after TPE, indicative of clinical responsiveness [21]. Collectively, these investigations advocate for TPE as a promising and well-tolerated adjunctive therapy for pediatric ADEM patients exhibiting inadequate responses to conventional treatments like steroids and IVIG. TPE emerges as a therapeutic avenue that can enhance neurological outcomes and facilitate functional recovery in pediatric ADEM cases [20,21].

Methodologies and Outcomes

The methodologies and outcomes of TPE in pediatric ADEM patients are predominantly derived from case reports and small-scale studies. These investigations collectively suggest that TPE represents an effective and safe therapeutic option for pediatric ADEM patients exhibiting inadequate responses to first-line treatments such as corticosteroids and IVIG [20]. A retrospective study scrutinized five pediatric ADEM cases where TPE was initiated following failed improvement with steroids and IVIG [13]. Predominant neurological symptoms encompass altered consciousness, seizures, motor deficits, cranial nerve disorders, and aphasia. Patients underwent four to five TPE sessions without encountering adverse effects. Notably, all patients exhibited progressive clinical amelioration, marked by enhanced responsiveness to stimuli, cessation of seizures, and restoration of mobility. At follow-up, one patient persisted with right-sided paresis, while another experienced ongoing epileptic seizures, yet neither reported new demyelinating episodes [13].

Additionally, a case report detailed the experience of a steroid- and IVIG-refractory ADEM patient who responded favorably to TPE, showcasing significant clinical improvement [22]. The authors concluded that TPE emerges as a favorable therapeutic avenue for pediatric ADEM, mainly when conducted at proficient centers, based on its tolerability and the observed neurological enhancements across their cases [13]. Furthermore, a broader study encompassing 18 children afflicted with various neuroimmunological diseases, including one ADEM case, underscored the immediate clinical improvement observed in 95% of patients post-TPE initiation, with 78% demonstrating significant improvement during follow-up [21]. Notably, only one patient diagnosed with optic neuritis exhibited resistance to TPE intervention [21]. While further research remains imperative, extant evidence indicates that TPE represents a potent and well-tolerated adjunctive therapy for pediatric ADEM patients, demonstrating suboptimal responses to initial treatments [13,21]. Notably, when executed at proficient centers, TPE tends to yield neurological enhancement across most cases [13,21].

Limitations and Critiques

The studies available on TPE in pediatric ADEM primarily consist of retrospective case series or reports with small sample sizes [20,21]. More extensive prospective studies are warranted to better evaluate the efficacy and safety of TPE in this population. Currently, TPE protocols lack standardization, including variability in the number of sessions, volume exchanged, and replacement fluids utilized [20,21]. Determining optimal TPE parameters specific to pediatric ADEM remains crucial for further investigation. Moreover, direct comparisons between TPE and other second-line therapies, such as IVIG or cyclophosphamide, need to be included in the existing literature [20,21]. Head-to-head trials are essential to ascertain whether TPE offers superior benefits to alternative treatments. Additionally, long-term follow-up data are limited, necessitating further research to evaluate the durability of clinical improvements achieved with TPE and the risk of relapse [20]. Furthermore, the heterogeneity of studies, encompassing patients with various acute demyelinating syndromes alongside ADEM, poses a challenge in drawing specific conclusions about TPE's efficacy and safety in the ADEM population [21]. More extensive ADEM-specific studies are warranted to provide more targeted insights. Additionally, inconsistent reporting of adverse events across studies underscores the need for systematic monitoring and reporting to comprehensively characterize the safety profile of TPE in children [20,21]. Despite these limitations, the available evidence suggests that TPE can serve as a valuable adjunct therapy for pediatric ADEM patients who do not respond adequately to first-line treatments [20,21]. Nevertheless, more extensive prospective studies are indispensable to confirm its efficacy and safety in this population.

Comparative analysis

Comparison of TPE with Other Treatment Modalities

A meta-analysis investigating TPE versus conventional treatment in the management of hypertriglyceridemia-induced acute pancreatitis (HTG-AP) revealed that TPE was superior in reducing triglyceride levels and improving clinical outcomes [23]. Additionally, a comparative study directly comparing membrane filtration (membrane therapeutic plasma exchange (mTPE)) and centrifuge-based (centrifugal therapeutic plasma exchange (cTPE)) TPE modalities found that while both methods removed similar amounts of plasma, cTPE boasted a significantly shorter total treatment time compared to mTPE (107 vs. 116.5 minutes, respectively) [24]. Despite this, both modalities exhibited comparable plasma removal efficiency rates, indicating that mTPE is as effective as cTPE (86.8% vs. 85.15%, respectively) [24]. A review article published in the American Journal of Kidney Diseases delineated various apheresis modalities targeting specific molecules. TPE, executed through centrifugation or membrane filtration, emerged as an extracorporeal therapy capable of isolating plasma from the blood and exchanging it with replacement fluids [25]. The accumulated evidence suggests that regardless of the method employed, TPE proves more efficacious than conventional treatment in managing specific conditions such as HTG-AP [23]. While both TPE modalities exhibit similar plasma removal efficiency, centrifugation-based TPE may offer a faster treatment option than membrane filtration [24].

Assessing the Role of TPE in the Context of Current Therapeutic Guidelines

The role of TPE in managing a spectrum of neurological and systemic diseases is substantial, with well-established efficacy demonstrated in conditions such as GBS and anti-N-methyl-D-aspartate (NMDA) receptor encephalitis. However, the utilization of TPE in MG lacks robust support from large-scale clinical studies, resulting in discrepancies between guidelines and real-world clinical practice, particularly in MG, where its use is predominantly off-label [26]. Several factors impede TPE's widespread adoption, including limited familiarity with the procedure, inadequacy of specialized facilities, and challenges related to venous access [26]. TPE modulates immune mechanisms by eliminating autoantibodies, immune complexes, and cytokines and influencing cellular components such as lymphocyte subsets [26]. Research indicates that TPE can foster the induction of T suppressor cells and alter the balance of T-helper type-1 (Th1) and type-2 (Th2) cells, pivotal in the pathogenesis of autoimmune disorders [27]. In pediatric neuroimmunological diseases, TPE has exhibited favorable clinical responses, with immediate improvement observed in 95% of cases and significant enhancement during follow-up in 78% of patients. The procedure is safe, with minimal complications reported [13]. Aligned with current therapeutic guidelines, TPE assumes a primary treatment role in diseases like GBS and NMDAR-related autoimmune encephalitis while serving as a secondary therapeutic option in conditions such as ADEM, MS, and NMOSD [25]. Nonetheless, further research is imperative to delineate optimal TPE regimens, encompassing parameters like duration, frequency, and cessation criteria, given the notable variability among different treatment centers [25,27]. Despite showing promise across various neurological and systemic diseases, ongoing research endeavors are crucial to deepen our understanding of TPE's therapeutic effects, refine treatment protocols, and ensure its seamless integration into clinical practice guided by existing therapeutic guidelines [25-27].

Future directions

Potential Areas for Further Research

Several avenues for further research in pediatric ADEM and TPE warrant exploration. Long-term follow-up studies are essential to comprehensively understand the outcomes of pediatric ADEM patients treated with TPE. These studies should focus on factors like relapse rates, the emergence of chronic neurological deficits, and quality of life measures [20]. Comparative effectiveness studies comparing TPE with other second-line therapies, such as cyclophosphamide or rituximab, in pediatric ADEM patients who do not respond to initial treatments like steroids and IVIG are imperative to delineate the optimal treatment approach [21]. Biomarker research holds promise in identifying predictive markers for TPE response in pediatric ADEM patients. This research could facilitate treatment decision-making and the implementation of personalized medicine approaches [21]. Exploration of optimal treatment strategies, including TPE sessions' timing, frequency, and duration, is crucial to maximize clinical improvement and minimize neurological deficits in pediatric ADEM. Randomized controlled trials could shed light on these optimal strategies [20]. Expanding the application of TPE to other pediatric acute demyelinating syndromes beyond ADEM, such as NMOSD and myelin oligodendrocyte glycoprotein antibody-associated disease, would provide insights into its efficacy in broader CNS inflammatory disorders [21]. Developing standardized treatment algorithms is essential to guide clinicians in appropriately using TPE in pediatric ADEM. These algorithms should consider disease severity, response to initial therapies, and neuroimaging findings to optimize patient outcomes [28].

Considerations for Optimizing TPE Protocols in Pediatric ADEM

Several research avenues in pediatric ADEM and TPE are worth exploring further. Firstly, long-term follow-up studies are crucial to comprehensively understanding the outcomes of pediatric ADEM patients treated with TPE. These studies should investigate factors such as relapse rates, chronic neurological deficits' emergence, and quality of life measures [29]. Secondly, comparative effectiveness studies comparing TPE with other second-line therapies, such as cyclophosphamide or rituximab, in pediatric ADEM patients who do not respond to initial treatments like steroids and IVIG are imperative to discern the optimal treatment approach [21]. Moreover, biomarker research holds promise in identifying predictive markers for TPE response in pediatric ADEM patients. Such research could aid treatment decision-making and the implementation of personalized medicine approaches [21]. Exploring optimal treatment strategies, encompassing TPE sessions' timing, frequency, and duration, is critical to maximizing clinical improvement and minimizing neurological deficits in pediatric ADEM. Randomized controlled trials could provide insights into these optimal strategies [30]. Additionally, expanding the application of TPE to other pediatric acute demyelinating syndromes beyond ADEM, such as NMOSD and myelin oligodendrocyte glycoprotein antibody-associated disease, could offer insights into its efficacy in broader CNS inflammatory disorders [31]. Lastly, developing standardized treatment algorithms is essential to guide clinicians in appropriately using TPE in pediatric ADEM. These algorithms should consider disease severity, response to initial therapies, and neuroimaging findings to optimize patient outcomes [21].

## Conclusions

In conclusion, this comprehensive review underscores the potential of TPE as a valuable therapeutic avenue for pediatric ADEM. By analyzing existing literature, it becomes evident that TPE holds promise in mitigating inflammation, improving neurological outcomes, and reducing long-term disability in affected children. However, the efficacy of TPE appears contingent upon various factors, including disease severity, timing of intervention, and patient-specific characteristics. The implications for clinical practice are substantial, suggesting clinicians should consider TPE early, particularly in severe or refractory cases where conventional treatments have proven insufficient. Moreover, interdisciplinary collaboration among healthcare professionals is paramount for optimizing treatment strategies and ensuring comprehensive care for pediatric ADEM patients. Looking ahead, future research endeavors should prioritize long-term prospective studies to assess treatment durability and neurodevelopmental outcomes. Additionally, comparative effectiveness trials and standardization of TPE protocols are essential for refining treatment algorithms and minimizing associated risks. Ultimately, sustained innovation and investigation in managing pediatric ADEM are critical for enhancing outcomes and quality of life for affected children and their families.
